# Competence in metered dose inhaler technique among dispensers in Mekelle

**DOI:** 10.1186/1710-1492-10-18

**Published:** 2014-04-23

**Authors:** Hussen D Ali, Gubena S Worku, Asfaw A Alemayehu, Woldearegay H Gebrehiwot

**Affiliations:** 1 Department of Pharmacy, College of Health Sciences, Mekelle University, Mekelle, Ethiopia; 2 Pharmacy unit, Minister of Defence Armed Force Referral Hospital, Health main directorate, Addis Ababa, Ethiopia; 3 Department of Midwifery, College of Health Sciences, Mekelle University, Mekelle, Ethiopia

**Keywords:** MDI, Asthma, Dispensers, Inhalers

## Abstract

**Background:**

Inhaled medications are the cornerstone of asthma therapy. Metered dose inhaler technique is a widely used technique to administer medications like corticosteroids. Meanwhile, the health professionals and patients knowledge and practice towards this metered dose inhaler is quite deficient but arguably understood by policy makers or education expertise.

**Objective:**

This study tried to assess the pharmacists and druggists competency on MDI who are the professionals at the front line to demonstrate and teach the technique for patients.

**Method:**

A cross sectional study was conducted among registered pharmacists and druggists from different public and private pharmacies and drug stores in Mekelle Town, Ethiopia from March to June, 2013. Evaluation tool was adapted from the National Asthma Education and Prevention Programmes of America (NAEPP) step criteria for the administration of a metered dose inhaler to score the knowledge/proficiency of use of MDIs by the subjects using two evaluators.

**Result:**

The mean score given by evaluators was 4.34 and 4.28 by evaluator I and II respectively. Of the 106 professionals took part in this research, based on the competency on essential steps for optimum therapeutic value of MDI, only 2 (1.9%) and 1 (0.9%) study participants had adequate competency in metered dose inhaler according to evaluator I and evaluator II respectively. The rest, irrespective of their age, sex, educational status and experience, did not achieve adequate score on MDI technique. Of the essential steps, only 25 (23.6%) and 16 (15.1%) participants breathed in and actuating the canister together according to evaluators I and II respectively.

**Conclusion:**

Very poor MDI technique was very common in this sample of healthcare providers. Despite involvement of all participants in patient counselling on inhalers, none of them were able to perform all steps correctly, which shows that patient may not have adequate instruction.

## Background

Asthma affects 235 million people today and the prevalence is rising [1]. Asthma is one of the top 20 causes of death in developing countries. In Ethiopia the prevalence of asthma accounted 9.9%. This is consistently increasing in sub Saharan countries particularly in urban regions. According to the latest WHO data published in April 2011 Asthma deaths in Ethiopia reached 7,309 or 0.89% of total deaths. The age adjusted Death Rate was 18.42 per 100,000 of population that puts Ethiopia rank 25^th^ in the world [1,2].

Currently two inhaler medications (beclomethasone and salbutamol) are available in all private, national procurement center and public hospital pharmacies in Ethiopia [1]. Appropriate management is required to improve asthma. For such reasons; use of inhalational corticosteroids are remarkably higher. One of the commonest delivery system for those inhalational drugs is Metered Dose Inhaler (MDI) [3]. Hence knowledge of such delivery method is mandatory for all prescribers, dispensers and consumers.

Poor control of asthma is mainly related to under diagnosis and inappropriate or inadequate management. The contribution of poor understanding of patients about the disease and treatment, non-adherence and incorrect use of devices are innumerable [4].

There have been many delivery systems developed for inhaled antiasthma medication, each with advantages and disadvantages. Among these, the most frequently used devices is metered dose inhaler (MDI), good inhaler technique and adequate adherence are important. With regard to technique, specific steps and good coordination are necessary for the proper use of this device. A less than optimal technique can result in decreased drug delivery and potentially reduced efficacy [5]. Even though Metered dose inhalers are widely used among Asthma patients; many of patients have difficulty in utilizing the device. Health care providers play a pivotal role in imparting a correct knowledge and use of MDI technique. But the reverse is true in most studies which seem to have suboptimal knowledge and skill on metered dose inhaler technique. It is evident that if health care providers are unable to demonstrate MDI technique correctly, educating patients would be ineffective [6-9]. In most cases, it is the pharmacist’s responsibility to ensure that patients know how to make the best use of their medication. With this assumption this study seeks to determine the practice of proper MDI technique among registered pharmacist and druggists in Mekelle.

## Methods

A cross sectional study was conducted among registered pharmacists and druggists from different public and private pharmacies and drug stores in Mekelle Town, Ethiopia from March to June, 2013. Evaluation tool was adapted from the National Asthma Education and Prevention Programmes of America (NAEPP) step criteria for the administration of a metered dose inhaler to score the proficiency of use of MDIs by the subjects [10]. Study subjects were given an MDI devise and asked to demonstrate the technique. During demonstration the dispensers were told to demonstrate the technique as if they are telling to the patient facing one individual that act as patient. Two pharmacists who obtained a special training on MDI technique tried to evaluate and give score for the given 11 criteria (Table 1). Scores were classified as correctly demonstrated, incorrectly demonstrated and skipped steps. Adequacy of inhalational techniques was demonstrated based on their ability to demonstrate all the essential steps (step one, Two, five, six, seven and Eight) and a total score of ≥ seven and those who were not demonstrating all the essential steps correctly and scores less than seven were considered as having poor competency in inhalational technique. Each pharmacist was evaluated individually and no oral instructions, prompts or critique was provided about inhalational techniques prior to or during the observations. But written consent has been obtained from each participant. In addition ethical clearance has been taken from Mekelle University, college of health sciences, institutional ethical review committee.

**Table 1:** Steps in using metered dose inhaler device adapted from the National Asthma Education and Prevention Programmes of America (NAEPP)

**Steps**	**Action performed**
1*	Shake the contents well
2*	Remove the cap
3	Hold the inhaler upright
4	Tilt the head back slightly
5*	Breath out slowly
6*	Open mouth with inhaler 1 to 2 inches away or in the mouth with the lips tightly sealed around it
7*	Begin breath in slowly and deeply through the mouth and actuate the canister once
8*	Hold breath for 10–20 sec
9	Exhale & wait one minute before the second dose
10	Shake again before the second dose
11	After use, replace the mouth piece cover

*Essential steps.

Data entry was done using *epiinfo* version 3.5.1 and data was transferred to SPSS version 16 and analyzed. Practice values were categorized by data transformation tool into two groups as poor and adequate based on the final scores. Finally inter observer agreement was determined using kappa statistics and interpreted according to Viera, and Garrett, 2005 [11].

## Result

A total of 113 pharmacists and druggist were approached and consented to participate in the study. Among these, 68 male and 38 female were willing to participate in the study and 7(6.2%) of the participants were not interested after the consent was given (Table 2). The median years of experience of the respondents was 3.5 years with a range of 0.5 to 36 years of which 25% were 2 years and below where as 75% were ≤7 years of experience.

**Table 2:** Socio demographic characteristics of respondents, March, 2013, Mekelle, Ethiopia

**Demographic characteristics**	**Parameter**		**Frequency (%)**
**Sex**	Male		68(64.2)
	Female		38(35.8)
**Age (in years)**	21-29		68(64.2)
	30-39		22(20.8)
	40-49		11(10.4)
	≥50		5(4.7)
**Category**	Pharmacist	1^st^ degree	53(50.0)
		Master	4(3.8)
	Druggist	Druggist	49(46.2)

Pharmacists and druggists competence in using correct inhaler technique was found very appalling. Both evaluators’ result showed poor demonstration technique. The mean score given by evaluator I was 4.34 ± 1.427 with a range of score 7 between the highest and the lowest scores. 95.3% of the respondents got score below 7. Similarly; the minimum and maximum score in the second evaluator was 0 and 9 respectively with mean score of 4.28 ± 1.835 (Table 3). 90.6% of same respondents scored below 7 according to judgment made by evaluator II. Kappa statistics shows a fair agreement between two observers with a value of 0.36. Meanwhile the two evaluators have Substantial agreement on those individuals who had adequate competency on the essential steps with a kappa of 0.66.

**Table 3:** Total score of the respondent for MDI technique, March, 2013, Mekelle, Ethiopia

**Evaluator 1**		**Evaluator 2**	
**Score**	**Frequency of respondent**	**Score**	**Frequency of respondent**
**0**	0	0	3
**1**	3	1	6
**2**	8	2	9
**3**	16	3	12
**4**	30	4	25
**5**	28	5	27
**6**	16	6	14
**7**	3	7	7
**8**	2	8	1
**9**	0	9	2

Five respondents as to evaluator I and 10 respondents from evaluator II maintain seven and above score. But only one individual according to evaluator I and two study participants as to evaluation made by evaluator II achieved adequate scores irrespective of the variation in Age, sex, Educational status and experience of study participants. Of those essential steps, step 7 (Begin breath in slowly and actuate the canister once) was the least correctly responded step. In contrary to that; step two ‘Remove the cap’ (88.7%) was most correctly demonstrated essential step followed by step 1 ‘Shake the contents well’ (64.2%) (Table 4).

**Table 4:** Frequency of evaluation of respondents to demonstrate each step of metered dose inhaler technique, March, 2013, Mekelle, Ethiopia

	**Evaluator 1**			**Evaluator 2**		
**Steps**	**Correct**	**Incorrect**	**Skipped**	**Correct**	**Incorrect**	**Skipped**
	**N (%)**	**N (%)**	**N (%)**	**N (% )**	**N (%)**	**N (%)**
1*	68(64.2)	3(2.8)	35(33)	74(69.8)	2(1.9)	30(28.3)
2*	94(88.7)	1(0.9)	11(10.4)	83(78.3)	2(1.9)	21(19.8)
3	48(45.3)	8(7.5)	50(47.2)	62(58.5)	10(9.4)	34(32.1)
4	21(19.8)	11(10.4)	74(69.8)	16(15.1)	8(7.5)	82(77.4)
5*	42(39.6)	4(3.8)	60(56.6)	31(29.2)	11(10.4)	64(60.4)
6*	48(45.3)	11(10.4)	47(44.3)	54(50.9)	13(12.5)	39(36.8)
7*	25(23.6)	12(11.3)	69(65.1)	16(15.1)	17(16)	73(68.9)
8*	34(32.1)	19(17.9)	53(50)	32(30.2)	16(15.1)	58(54.7)
9	10(9.4)	21(19.8)	75(70.8)	11(10.4)	19(17.9)	76(71.7)
10	5(4.7)	16(15.1)	85(80.2)	17(16)	12(11.3)	77(72.6)
11	65(61.3)	1(0.9)	40(37.7)	58(54.7)	1(0.9)	47(44.3)

*The essential steps.

## Discussion

The introduction of metered dose inhalers is a major innovation in the therapeutic management of bronchial asthma and chronic obstructive airway disease. These devices enable the direct delivery of medication to the respiratory system, hence reducing the first pass effect while minimizing systemic side effects. However, as only 8.8% of the aerosolized dose reaches the small conducting airway and alveoli even with the proper use of MDIs, it is important that the pharmacist performs the MDI technique correctly. Pharmacists and druggists have responsibility to ensure that patients use prescribed medications correctly. But, this cannot be achieved when those who teach patients have questionable skills [7]. Of the 106 professionals took part in this research, one (0.94%) individual based on evaluation made by evaluator I and two participants as to evaluator II had adequate competency in metered dose inhaler. This result indicated far lower than studies from both developed and developing countries [8,9]. In addition, no respondent has got all the steps right which is lower than study from Oman where 15% of the respondents performed all the steps correctly. The problem with effective demonstration skill could be the principle in higher education more focused on knowledge and attitude development rather than pressing on skills. Moreover, it signifies that most dispensers are simply instructing patients without actual demonstration. As it has been observed in the methodology before beginning the observational evaluation written consent have been obtained. Even though they already know that they are under investigation, poor results have been obtained. Had it been without the knowledge of the study subject; their true competency could even worse than what has been found.

Study result shows a serious action on education of asthma inhaler and patient care. Because, it is difficult for patients to read and understand manufacturers label where the medium of communication is quite different from speaking language of the community. Effective patient education improves the patient care safety and brings positive healthcare outcomes [9]. Most importantly, authors would like to stress on routine intervention or trainings concerning instructions and demonstration of the inhaler technique could improve the skills among health practitioners as it has been demonstrated from other research [12].

Each step is designed in a way that optimum bioavailability would be achieved from administration. If a patient misses one of the essential steps or the steps needed for adequate therapy, it ends up with compromise in therapeutic success. During patient education, dispensers should emphasize at least on the essential steps. However, only two individuals according to evaluator II and one participant as to the evaluation made by evaluator I gave high attention to the essential steps. Reprehensibly, step 7 (Begin breath in slowly and actuate the canister once) was the most skipped (65.1%) essential step according to both evaluators. These values indicate that both evaluators were unanimous on the result of step 7. Similarly depressing the canister was with high frequency of error according a study from Iran. In addition to this; ‘Shake again before the second dose’ was least correct response among non-essential but preferred steps. While inadequate breath-holding and waiting before a second puff practiced among health care providers were with high frequency of error in Iran and Oman study [2,13]. In both cases, whether step 7 or 8; could lower the optimum therapeutic effect of MDI if skipped or incorrectly administered.

In addition, the result from both evaluators agreed on highest score received was removing the cap (step 2). The first step during MDI administration was ‘shake the content’ in order to keep uniformity of delivered dose. Unless prescribed in the instructions, patients have to shake the inhaler for 5 seconds. But, in our study more than 30% of respondents were not able to demonstrate it or skipped. Next to step 10 (Shake again before the second dose); most skipped and incorrectly demonstrated step was Exhale & wait one minute before the second dose with 9.4% and 10.4% correct demonstration from evaluator I and II respectively. Similar result was observed from Napalese teaching hospital with 9.79% upon pre-intervention assessment [3].

A number of factors have been issued on inadequate patient education on asthma inhalers. Among these, lack of regular periodic assessment of patients’ inhaler technique, lack of time for educating patients, and lack of awareness regarding the importance of patient education took the highest priority of concern. Even though most researches directed the possible solution to hospital administrators, the authors of this paper would like to reflect the idea to higher institutions and regulatory bodies since most of the research participants were from the private pharmacy and drug shops.

Different studies have shown trainings involving instructions and demonstration of the inhaler technique have been shown to improve the skills of patients and providers. Very poor inhaler technique observed in our study is most probably due to the lack of any formal training for healthcare providers on the correct use of inhalers. Studies have shown that patients did not receive formal education by health care professionals regarding the proper use of inhaler devices. The argument they brought is lack of asthma education programme [14].

## Conclusion and recommendation

Finding raises concerns as it is the single most important step of the whole inhalation technique since lesser amounts of the inhaled drug would reach the lungs, which would result in poor control of asthma. Results of this study showed that very poor MDI technique was very common in this sample of healthcare providers. Despite involvement of all participants in patient counselling on inhalers, none of them were able to perform all steps correctly, which shows that patient may not have adequate instruction.

In conclusion, healthcare providers’ skill in the MDI technique among pharmacists and druggists in Mekelle town is very limited, indicating the need for establishing regular educational programs for health care providers [6,7,12]. Associations, higher education’s, governmental and nongovernmental organizations should take part in resolving the problem. Moreover, the authors suggest research on the effect of health professionals dispensing advice on patients’ treatment outcome. Therefore, it is essential that educational institutions must look into their curricula and quality education that matters to the community. Moreover, regulatory bodies should have a routing follow-up in those service delivery organizations and assure the quality of services provided through competency measurement in the respected areas; especially chronic illnesses which are currently dominating the list of top killer diseases in the world. Furthermore, continuous reinforcement among the professionals is necessary to make them realize the importance of asthma education and training which will ultimately enhance the patient care quality and their satisfaction [9].

## Competing interests

There is no financial and non financial competing interest among authors.

## Authors’ contributions

GSW have made contribution starting from generating idea to data collection. While HDA responsible in designing the study, supervising the research and involved in drafting the manuscript. WHG and AAA participated in the acquisition of data analysis and interpretation. Finally all authors read and approved the manuscript for publication.
